# Opportunities and Challenges for Molecular Understanding of Ciliopathies–The 100,000 Genomes Project

**DOI:** 10.3389/fgene.2019.00127

**Published:** 2019-03-11

**Authors:** Gabrielle Wheway, Genomics England Research Consortium, J. C. Ambrose, Hannah M. Mitchison

**Affiliations:** (1) Genomics England, London, UK; (2) William Harvey Research Institute, Queen Mary University of London, London, EC1M 6BQ, UK; ^1^Human Development and Health, Faculty of Medicine, University of Southampton, Southampton General Hospital, Southampton, United Kingdom; ^2^Genetics and Genomic Medicine, University College London, UCL Great Ormond Street Institute of Child Health, London, United Kingdom

**Keywords:** 100,000 Genome Project, ciliopathies, cilia, genomics, genetics

## Abstract

Cilia are highly specialized cellular organelles that serve multiple functions in human development and health. Their central importance in the body is demonstrated by the occurrence of a diverse range of developmental disorders that arise from defects of cilia structure and function, caused by a range of different inherited mutations found in more than 150 different genes. Genetic analysis has rapidly advanced our understanding of the cell biological basis of ciliopathies over the past two decades, with more recent technological advances in genomics rapidly accelerating this progress. The 100,000 Genomes Project was launched in 2012 in the UK to improve diagnosis and future care for individuals affected by rare diseases like ciliopathies, through whole genome sequencing (WGS). In this review we discuss the potential promise and medical impact of WGS for ciliopathies and report on current progress of the 100,000 Genomes Project, reviewing the medical, technical and ethical challenges and opportunities that new, large scale initiatives such as this can offer.

## The 100,000 Genomes Project

The launch of the UK's 100,000 Genomes project was announced in December 2012 as part of the UK's Life Sciences Strategy. This ambitious £300 million national project aimed to sequence 100,000 complete genomes from 70,000 individuals with cancer or rare disease, and their unaffected family members (Turnbull et al., 2018). Unlike other population genomics studies such as those in Iceland, Japan, Finland, Sweden and the Netherlands (An, 2017), the 100,000 Genomes Project is a hybrid clinical/research initiative, with an aim to fully integrate genomic testing for eligible individuals within existing routine healthcare pathways in the UK National Health Service (NHS). Sequence data is linked to longitudinal patient records such as hospital admissions and responses to interventions, providing a rich resource of genomic medical information. In many respects, it is the first scheme of its kind in the world, and one of the largest.

The project built upon the legacy of successful population healthcare genetics studies in the UK, such as the Deciphering Developmental Disorders (DDD) study (Wright et al., 2015) and UK10K (Consortium et al., 2015) which built upon the Avon Longitudinal Study of Parents And Children (ALSPAC) (Boyd et al., 2013) and TwinsUK (Moayyeri et al., 2013). It aims to secure the UK's position as a world-leader in healthcare and genomics.

**Figure 1:**
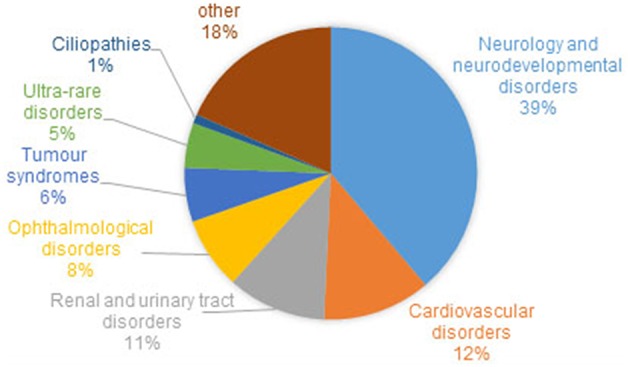
Proportion of families and individuals with different disorders within the rare disease cohort of the 100,000 Genomes Project. Around 1% of recruited families/individuals have a ciliopathy. Cohort summary data courtesy of Genomics England, with permission.

Recruitment for the 100,000 Genome Project has been coordinated across 13 Genomic Medicine Centers set up across 85 NHS Trusts in England, Northern Ireland and Scotland. Of the estimated 8,000 rare diseases (Boycott et al., 2017), 190 were recruited to the Rare Disease pathway of the 100,000 Genomes Project, from 2015/16 until 31st September 2018. Eligible rare diseases were nominated by clinicians and researchers who felt there was an unmet clinical need, for example, diseases for which there are a sizeable proportion of patients with no known genetic diagnosis. The range of phenotypes and the proportion of patients with different disorders within the rare disease cohort is shown in Figure 1. Where possible, “trios” were recruited, i.e., the affected individual and both parents, with efforts made to recruit both affected and unaffected family members to allow effective variant filtration.

## Genetic Basis of Ciliopathies—Diseases Affecting Cilia Structure And Function

Ciliopathies represent one group of rare diseases recruited to the 100,000 Genomes project. The ciliopathies are a diverse grouping but have a common etiology, originating in ciliogenesis (growth and maintenance) and/or structural or functional defects of the cilium (Reiter and Leroux, 2017). Cilia are highly evolved, complex organelles with important roles in motility and signaling (Waters and Beales, 2011; Mitchison and Valente, 2017; Wheway et al., 2018). The extent of the overall medical impact of ciliopathies, which individually most often are rare conditions but collectively affect a large number of individuals, has emerged over the last 20 years as their functions have continued to be revealed through genetic analysis in animal models and human families (Fliegauf et al., 2007; Goetz and Anderson, 2010).

Cilia can broadly be divided into motile and non-motile (primary), each with separate functions, both of which form a unique cellular compartment distinct from the rest of the cell. Primary cilia are more ubiquitous, distributed across most cell types of the body, extending out from the cell surface into their external environment as a single projection with diverse mechanosensory and chemosensory signal transduction roles (Singla and Reiter, 2006). The photoreceptors of the eye have a modified cilium, the outer segment (Wright et al., 2010). In contrast, motile cilia are found only on specialized epithelial surfaces and usually as multiple motile cilia (perhaps 200 per cell). Motile cilia lining notably the respiratory airways, brain ventricles and fallopian tubes, move essential body fluids and gametes, with a structural composition related to that of sperm flagella in male gamete movement (Spassky and Meunier, 2017). Single motile nodal cilia that exist transiently in the embryonic left-right organizer function in laterality determination (Hamada, 2016).

Owing to the central role of the cilium in development and health, ciliopathies often include complex multi-organ developmental phenotypes. The most severe-lethal ciliopathies, Meckel-Gruber syndrome (MKS) (Wheway and Johnson, 2014; Hartill et al., 2017), Joubert syndrome (JBTS) (Parisi and Glass, 1993; Bachmann-Gagescu et al., 2015), orofacial digital syndrome (OFD) (Gurrieri et al., 2007) as well as Bardet-Biedl syndrome (BBS) (Forsythe and Beales, 2013), all include neurodevelopmental features. These are often present alongside other features such as retinal dystrophy, renal dysplasia and skeletal abnormalities (Waters and Beales, 2011). Skeletal ciliopathies range from lethal short-rib thoracic dysplasias to more mild phenotypes such as Jeune syndrome (Huber and Cormier-Daire, 2012; Mitchison and Valente, 2017; Schmidts and Mitchison, 2018). Renal ciliopathies are some of the most common conditions associated with cilia defects. Autosomal polycystic kidney disease (ADPKD) alone affects between 1:2,500 and 1:4,000 individuals in the EU, making it one of the most common genetic diseases in humans, and the most common cause of end–stage renal failure (Willey et al., 2017). Renal malformations can also take the form of nephronophthisis, characterized by chronic tubulointerstitial nephritis, in isolated conditions or part of multiorgan ciliopathies including Senior-Loken syndrome (Wolf and Hildebrandt, 2011). Due to the importance of the highly specialized photoreceptor cilium in the function of the retina, many syndromic ciliopathies include a retinal dystrophy phenotype. Furthermore, around one third of non-syndromic inherited retinal dystrophies, such as retinitiris pigmentosa (RP) and Leber congenital amaurosis (LCA), are associated with a retinal cilium defect (Estrada-Cuzcano et al., 2012). These are termed retinal ciliopathies (Bujakowska et al., 2017).

Rather than being distinct clinical entities, ciliopathies are considered to form a spectrum of disorders, with considerable phenotypic and genotypic overlap between different conditions. In addition to this, there is also extensive phenotypic and genetic heterogeneity amongst ciliopathies (Mitchison and Valente, 2017; Oud et al., 2017). There are currently at least 190 known ciliopathy disease genes causing defects of the primary cilia (Table 1), and mutations in many of the same genes can cause strikingly different phenotypes. A classic example is *CEP290*, mutations in which can cause perinatal lethal MKS, severe JBTS, or non-syndromic LCA, which only affects the retina (Coppieters et al., 2010; Drivas and Bennett, 2014).

**Table 1:** Overview of genes mutated in ciliopathies showing the heterogeneity of certain ciliopathies.

**Gene**	**OMIM**	**Description**
**NEURODEVELOPMENTAL CILIOPATHIES**		
*AHI1*	608894	JBTS
*ARL3*	604695	JBTS, RP?
*ARL13B*	608922	JBTS
*ARMC9*	NA	JBTS
*ATXN10*	611150	JBTS-like
*B9D1*	614144	MKS
*B9D2*	611951	MKS
*C5orf42*	614571	JBTS, OFDS
*CC2D2A*	612013	JBTS, MKS
*CELSR2*	604265	JBTS-like
*CEP104*	616690	JBTS
*CEP120*	613446	JBTS, SRTD
*CEP290*	610142	JBTS, SLSN, LCA, MKS, BBS
*CEP41*	610523	JBTS
*CEP55*	610000	MKS-like
*CLUAP1*	616787	JBTS, OFDS
*CPLANE1*	614571	JBTS
*CSPP1*	611654	JBTS
*EXOC4*	608185	MKS
*EXOC8*	615283	JBTS
*HYLS1*	610693	MKS-like
*ICK*	612325	ECO
*IFT81*	605489, 617895	JBTS-like, SRTD
*INPP5E*	613037	JBTS
*KIAA0556*	616650	JBTS
*KIAA0586*	610178, 616546	JBTS, SRTD
*KIF14*	611279	MKS, microcephaly with kidney defects
*KIF7*	611254	JBTS, MKS-like, ACLS
*MKS1*	609883	MKS, BBS, JBTS
*NPHP1*	607100	JBTS, NPHP, SLSN
*NPHP3*	608002	MKS, NPHP
*OFD1*	300170	JBTS, OFDS
*PDE6D*	602676	JBTS
*PIBF1*	607532	JBTS
*RPGRIP1L*	610937	JBTS, MKS
*SUFU*	607035	JBTS
*TCTN1*	609863	JBTS
*TCTN2*	613846	JBTS
*TCTN3*	613847	JBTS, OFDS
*TMEM107*	616183	MKS, OFDS
*TMEM138*	614459	JBTS
*TMEM216*	613277	JBTS, MKS
*TMEM231*	614949	JBTS, MKS
*TMEM237*	614423	JBTS
*TMEM67*	609884	MKS, JBTS, NPHP, BBS
*TTBK2*	611695	JBTS-like
*ZNF423*	604557	JBTS, NPHP
**CILIOPATHIES WITH MAJOR SKELETAL INVOLVEMENT**		
*C2CD3*	615944	OFDS
*CEP120*	613446	SRTD
*C21ORF2*	603191	JATD
*DDX59*	615464	OFDS
*DYNC2LI1*	617083	SRTD
*DYNC2H1*	603297	JATD, SRTD
*EVC*	604831	EVC, WAD
*EVC2*	607261	EVC, WAD
*IFT122*	606045	CED
*IFT140*	614620	SRTD, RP
*IFT172*	607386	JATD, MZSDS, SRTD, RP
*IFT43*	614068	CED, SRTD, RP
*IFT52*	617094	SRTD +/− polydactyly +/− LCA
*IFT80*	611177	JATD, SRTD +/− retinal dystrophy
*INTU*	610621	OFDS? SRTD + polydactyly?
*KIAA0753*	617112	OFDS? JBTS?
*NEK1*	604588	SRTD
*OFD1*	300170	OFDS, SGBS, JBTS
*SCLT1*	611399	OFDS
*TBC1D32*	615867	OFDS
*TCTEX1D2*	617353	JATD, SRTD
*TTC21B*	612014	JATD, NPHP
*WDR19*	608151	JATD, CED, NPHP, SLSN
*WDR34*	613363	JATD, SRTD
*WDR35*	613602	EVC, CED, SRTD
*WDR60*	615462	JATD, SRTD
**ISOLATED AND SYNDROMIC OBESITY**		
*ALMS1*	606844	ALMS
*ARL6*	608845	BBS, RP
*BBIP1*	613605	BBS
*BBS1*	209901	BBS
*BBS10*	610148	BBS
*BBS12*	610683	BBS
*BBS2*	606151	BBS, RP
*BBS4*	600374	BBS
*BBS5*	603650	BBS
*BBS7*	607590	BBS
*BBS9 (PTHB1)*	607968	BBS
*C8orf37*	614477	BBS, CORD, RP
*CCDC28B*	610162	BBS
*CEP19*	615586	MOSPGF
*IFT27*	615870	BBS, unclassified lethal ciliopathy with renal involvement
*IFT74*	608040	BBS
*LZTFL1*	606568	BBS
*MKKS*	604896	BBS, MKKS
*TRIM32*	602290	BBS
*TTC8*	608132	BBS, RP
*WDPCP*	613580	BBS
**RENAL CILIOPATHIES**		
*ANKS6*	615370	NPHP
*CEP164*	614848	NPHP
*CEP83*	615847	NPHP
*DCDC2*	605755	NPHP
*GLIS2*	608539	NPHP
*INVS*	243305	NPHP
*IQCB1*	609237	SLSN
*MAPKBP1*	616786	NPHP
*NEK8*	609799	NPHP
*NPHP4*	607215	NPHP, SLSN
*PKD1*	601313	ADPKD
*PKD2*	173910	ADPKD
*PKHD1*	606702	ARPKD
*SDCCAG8*	613524	SLSN, BBS
*TRAF3IP1*	607380	SLSN
*VHL*	608537	VHL
*XPNPEP3*	613553	NPHP-like
**ISOLATED RETINAL CILIOPATHY**		
*C21orf71*	613425	RP
*CDHR1*	609502	RP, CORD
*CLRN1 (USH3A)*	606397	RP, USH
*EYS*	612424	RP
*LCA5*	611408	LCA
*MAK*	154235	RP
*PRPF3*	607301	
*PRPF31*	606419	RP
*PRPF4*	607795	RP
*PRPF6*	613979	RP
*PRPF8*	607300	RP
*RP1*	603937	RP
*RPGR*	312610	RP, PCD
*RPGRIP1*	605446	LCA
*SNRNP200*	601664	RP
*SPATA7*	609868	RP, LCA
*TOPORS*	609507	RP
*TULP1*	602280	RP, LCA
*USH2A*	608400	RP, USH
**RETINAL DYSTROPHY WITH SENSORINEURAL HEARING LOSS**		
*ADGRV1*	602851	USH
*ARSG*	610008	USH
*CDH23*	605516	USH
*CEP78*	617110	CORD + deafness
*CIB2*	605564	USH
*HARS*	142810	USH
*MYO7A*	276903	USH
*PCDH15*	605514	USH
*PDZD7*	612971	USH
*SANS*	607696	USH
*TUBB4B*	602662	LCA with early-onset hearing loss
*USH1C*	605242	USH
*USH1E*	602097	USH
*USH1H*	612632	USH
*USH1K*	614990	USH
*WHRN*	607928	USH
**OTHER CILIOPATHIES**		
*CENPF*	600236	Microcephaly, agenesis of corpus callosum
*CCDC11*	614759	Laterality defects
*CDK10*	603464	Suspected complex multisystem ciliopathy affecting development, speech with agenesis of corpus callosum, sensorineural deafness, retinitis pigmentosa, vertebral anomalies, patent ductus arteriosus, facial dysmorphism
*SPAG17*	616554	Brain malformations, CED-like skeletal dysplasia
*WDR11*	606417	congenital hypogonadotropic hypogonadism, Kallmann syndrome
**PRIMARY CILIARY DYSKINESIA**		
*ARMC4*	615408	PCD
*C21orf59*	615494	PCD
*CCDC103*	614677	PCD
*CCDC114*	615038	PCD
*CCDC151*	615956	PCD
*CCDC39*	613798	PCD
*CCDC40*	613799	PCD
*CCDC65*	611088	PCD
*CCNO*	607752	PCD
*CFAP300*	618058	PCD
*DNAAF1*	613190	PCD
*DNAAF2*	612517	PCD
*DNAAF3*	614566	PCD
*DNAAF4*	608706	PCD
*DNAAF5*	614864	PCD
*DNAH1*	603332	Male infertility, PCD association
*DNAH11*	603339	PCD
*DNAH5*	603335	PCD
*DNAH9*	603330	PCD
*DNAI1*	604366	PCD
*DNAI2*	605483	PCD
*DNAJB13*	610263	PCD
*DNAL1*	610062	PCD
*DRC1*	615288	PCD
*GAS8*	605179	PCD
*HYDIN*	610812	PCD
*LRRC6*	614930	PCD
*LRRC56*	N/A	Mucociliary clearance and laterality defects
*MCIDAS*	614086	PCD
*MNS1*	610766	Male infertility, laterality defects
*NME8*	607421	PCD
*PIH1D3*	300933	PCD
*RSPH1*	609314	PCD
*RSPH3*	615876	PCD
*RSPH4A*	612647	PCD
*RSPH9*	612648	PCD
*SPAG1*	603395	PCD
*STK36*	607652	PCD
*TTC25*	617095	PCD
*ZMYND10*	607070	PCD

*Modified from Oud et al. (2017)*.

*ACLS, acrocallosal syndrome; ADPKD, autosomal dominant polycystic kidney disease; ALMS, Alström syndrome; ARPKD, autosomal recessive polycystic kidney disease; BBS, Bardet-Biedl syndrome; CED, cranioectodermal dysplasia syndrome; CORD, cone-rod dystrophy; ECO, endocrine-cerebro-osteodysplasia syndrome; EVC, Ellis-van Creveld syndrome; JATD, Jeune asphyxiating thoracic dysplasia; JBTS, Joubert syndrome; LCA, Leber congenital amaurosis; MKKS, McKusick-Kaufman syndrome; MKS, Meckel-Gruber syndrome; MOSPGF, morbid obesity and spermatogenic failure; NPHP, nephronophthisis; OFDS, oral-facial-digital syndrome; PCD, primary ciliary dyskinesia; RP, retinitis pigmentosa; SGBS, Simpson-Golabi-Behmel syndrome; SLSN, Senior-Løken syndrome; SRTD, short-rib thoracic dysplasia; USH, Usher syndrome; VHL, von Hippel-Lindau syndrome; WAD, Weyers acrodental dysostosis. OMIM: ID number in Online Mendelian Inheritance in Man (https://omim.org/). NA: OMIM number is not available*.

Motile ciliopathies are grouped under the name primary ciliary dyskinesia (PCD). A component of PCD manifests with left-right axis abnormalities and this association is also called Kartagener's syndrome, which affects around 50% of PCD patients; additionally there is a more rare disease component arising from defects of ciliogenesis affecting cilia numbers, which is also being called reduced generation of multiple motile cilia (RGMC) (Boon et al., 2014; Lucas et al., 2014; Knowles et al., 2016). Apart from laterality defects that may be linked to cardiac disease (Best et al., 2018), PCD disease features include chronic respiratory infections from earliest life, progressive upper respiratory problems and loss of lung function (bronchiectasis), conductive hearing problems, subfertility, and infrequent hydrocephalus. The same as for the nonmotile ciliopathies, PCD is a genetically and clinically heterogeneous condition, with mutations in around 40 different motile cilia genes currently recognized to cause disease (Table 1). Similarly to the primary ciliopathies, a wider than previously suspected spectrum of motile ciliopathy disease is starting to emerge with greater genetic understanding of these conditions. Gene mutations causing more severe (Davis et al., 2015; Amirav et al., 2016; Irving et al., 2018) and more mild (Knowles et al., 2014; Lucas et al., 2017b; Best et al., 2018; Irving et al., 2018; Shoemark et al., 2018) disease have more recently been recognized, as well as subtypes with features that overlap with the primary ciliopathies such as retinitis pigmentosa and developmental delay (Budny et al., 2006; Moore et al., 2006). These syndromic disease subtypes e.g. Simpson-Golabi-Behmel type 2 syndrome, expand our understanding of the extent of the motile ciliopathy disease spectrum (Mitchison and Shoemark, 2017).

## Challenges of Diagnosing Ciliopathies Using Genomics

Despite advances in genetic understanding of these conditions with the advent of next generation sequencing, ciliopathies remain under-diagnosed and poorly recognized due to insensitive and non-specific aspects of available diagnostic tests, compounded by variable disease features. Genetic diagnostic rates of severe primary ciliopathies remain around 62% using targeted gene panel sequencing (Bachmann-Gagescu et al., 2015; Knopp et al., 2015) and 44% using whole exome sequencing (Sawyer et al., 2016). The diagnostic rate of the arguably more uniform motile ciliopathies disease grouping is higher, using well-characterized cohorts, at up to 67% using targeted gene panels (Boaretto et al., 2016; Paff et al., 2018) and 76% using whole exome sequencing with targeted copy number variation (CNV) analysis (Marshall et al., 2015). For both the primary and motile ciliopathies, many genetic causes of these conditions remain unknown. There remain very few, if any, treatment options for the vast majority of these conditions (Lucas et al., 2014, 2017a; Molinari and Sayer, 2017). Thus, there is a pressing clinical need to advance genetic understanding for the purpose of diagnostics, prognostics and development of novel targeted therapies. Clinical genome data from the 100,000 Genomes Project presents exciting opportunities to offer patients genetic diagnoses, gain novel insights into etiology of disease, and uncover new targets for therapies.

Ciliopathy patients and family members account for around 1% of the rare disease cohort recruited to the UK 100,000 Genomes Project (Figure 1). In the majority of cases, patients recruited to the ciliopathy pathway have had the relevant ciliopathy disease genes sequenced and excluded as the cause of their disease. Such testing is provided as a genetic diagnostic service by accredited NHS genomics labs, and involves sequencing the exons and intron/exon boundaries of a panel of currently 123 genes known to be mutated in ciliopathies.

At the time of writing, genomes have been sequenced and analyzed from 274 patients and family members with respiratory ciliopathies (126 PCD patients, 148 non-cystic fibrosis (CF) bronchiectasis patients and family members) and 81 patients and family members with congenitial malformations caused by ciliopathies (45 BBS, 14 JBTS, 22 rare multisystem ciliopathy disorders). These numbers will increase as sequencing and analysis is ongoing. However, it is likely that a much larger number of ciliopathy patients have been recruited to the project within other categories such as “renal and urinary tract disorders,” which includes phenotypic descriptor “cystic kidney disease” which accounts for 1,516 individuals alone. Furthermore, within the “ophthalmological disorders” category there are likely to be many undiagnosed retinal ciliopathy patients. One thousand two hundred and sixty individuals with a diagnosis of rod-cone dystrophy or LCA)/early onset severe retinal dystrophy have been recruited. It can be estimated that around one third of these patients have a retinal dystrophy owing to a retinal photoreceptor cilium defect (Estrada-Cuzcano et al., 2012). There are many patients in the project with other dysmorphic and congenital abnormalities with features overlapping ciliopathy phenotypes, which may be undiagnosed ciliopathies. This includes 19 patients with unexplained monogenic fetal disorders. Similarly, there are more than 6,000 patients (39% of the total rare disease patient cohort, Figure 1) with a general neurology or neurodevelopmental disorder. These include patients with intellectual disability, holoprosencephaly and hereditary ataxia, all of which are ciliopathy phenotypes seen in syndromic ciliopathies. A subset of these patients could also have undiagnosed ciliopathies. Numbers will increase as data from more patients is made available in upcoming data releases. The 100,000 Genomes Project may thus represent a unique opportunity to discover and diagnose orphan ciliopathies in patients whose phenotype does not currently easily fit into existing disease categories.

The success of the project may lie in the accuracy and thoroughness of phenotyping information provided i.e., the consistent use of Human Phenotype Ontology (HPO) terms. The HPO system was developed to annotate clinical disease terms and definitions with a standardized phenotypic vocabulary (Robinson et al., 2008; Köhler et al., 2017). A particular difficulty with ciliopathies is that they are extremely heterogeneous conditions and it has been suggested that clinicians should be encouraged to actively involve patients in describing their own phenotype (Gainotti et al., 2018). There is great power in larger collections of well-defined patient cohorts to support better clinical research and diagnostics, with the development of rare disease patient registries being a key component supporting the activities of European Reference Networks (ERNs) on rare diseases (Kodra et al., 2018). The European Organization for Rare Diseases (EURORDIS) has developed recommendations on ethical and responsible international data sharing to help inform a clinical diagnosis (Gainotti et al., 2018).

**Figure 2:**
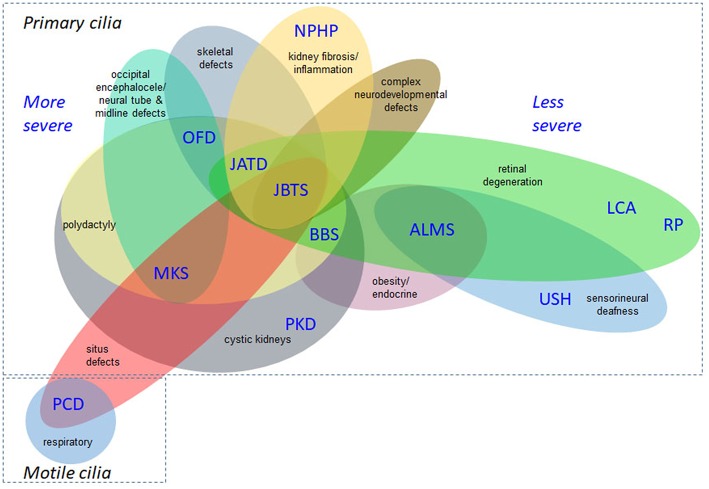
Overlapping disease features of the ciliopathies. This is not an exhaustive list but illustrates the complex phenotypes and overlapping clinical features of ciliopathies. The severe/lethal diseases tend to have more complex combinations of disease features, compared to diseases at the milder end of the clinical spectrum. Situs inversus and associated cardiac malformations are found in common between non-motile and motile ciliopathies and the former can also display respiratory defects. ALMS, Alström syndrome; BBS, Bardet-Biedl syndrome; JATD, Jeune asphyxiating thoracic dysplasia; JBTS, Joubert syndrome; LCA, Leber congenital amaurosis; MKS, Meckel-Gruber syndrome; NPHP, nephronophthisis; OFD, oral-facial-digital syndrome; PCD, primary ciliary dyskinesia; PKD, polycystic kidney disease; RP, retinitis pigmentosa; USH, Usher syndrome.

A wide range of phenotypes of different severities are associated with the ciliopathies, demonstrating their complexity (Lee and Gleeson, 2011; Arts and Knoers, 2013; Mitchison and Valente, 2017). Figure 2 is not exhaustive but shows selected clinical features of the ciliopathies, highlighting those relevant to recruitment criteria for 100,000 Genomes Project. This is a constantly expanding spectrum, with motile and non-motile cilia recently implicated in the etiology of congenital heart disease (You et al., 2015; Best et al., 2018). *Situs inversus* and associated cardiac malformations found in common between motile and some nonmotile ciliopathies suggest an influence on laterality determination at the embryonic left-right organizer during development from non-motile as well as motile cilia, or else that there are shared motile cilia defects, or a mixture of both. There is growing evidence for respiratory involvement in the non-motile primary ciliopathies, but the molecular basis of these findings remains unclear (Mitchison and Shoemark, 2017).

In terms of genetic heterogeneity, interpreting large volumes of genetic variants from all of the ciliopathy patients in order to identify the truly pathogenic disease-causing variant in each individual represents a major challenge. In addition to primary causal variants, modifier genes and variable mutational load have been proposed to play roles in determining the genetic spectrum for both primary ciliopathies (Katsanis et al., 2001; Davis et al., 2011; Zaki et al., 2011; Lindstrand et al., 2016) and motile ciliopathies (Li et al., 2016). Different mutation types can be expected in different ciliopathies, for example motile cilia disease tends to arise from high impact pathogenicity mutations, most often single base substitutions, small insertions and deletions or larger CNVs, that result in protein frameshifts, premature stop codons, missense changes, or splicing defects giving rise to null alleles effects. In contrast, for the non-motile primary ciliopathies lethality frequently arises as a consequence of such alleles, whilst surviving patients would carry only one or no copies of this type of high impact allele, but instead carry one or two hypomorphic alleles such as milder effect missense changes (Davis et al., 2011; Hildebrandt et al., 2011; Schmidts et al., 2013; Reiter and Leroux, 2017). Some specific mutations may be expected, for example splicing mutations and CNVs are common in retinal degeneration caused by *PRPF31* mutations (Buskin et al., 2018). With the superior detection of many mutations through whole genome sequencing, the genetic landscape is expected to greatly expand and may significantly change with implementation of large scale whole genome sequencing (Belkadi et al., 2015). For example the 100,000 Genome Project has already detected a deep intronic mutation in *DNAH11* causing PCD which would not have been detected by other current clinical screening methods (Ellingford et al., 2018).

## Ciliopathy Genomics Data Analysis in the 100,000 Genomes Project

Genomics England Clinical Interpretation Partnerships (GeCIPs) currently coordinate a crowdsourcing approach to data analysis, building on the success of aggregate consortia such as the Exome Aggregation Consortium (ExAc) (Karczewski et al., 2017) and Genome Aggregation Database (GnomAD) (Lek et al., 2016), and public databases such as ClinVar (Landrum and Kattman, 2018), Human Gene Mutation Database HGMD (Stenson et al., 2017) and the Leiden Open-source Variation Database LOVD (Fokkema et al., 2011). In addition to GeCIPs, formed of individuals from not-for-profit organizations, such as academics and clinicians who must apply for access to anonymized data, private commercial companies also have access to the anonymized data. The project is achieved through public-private partnerships (PPPs) between Genomics England Limited, owned by the UK Government's Department of Health & Social Care, and private companies including Illumina Inc. Illumina is a partner both in sequencing and bioinformatics data analysis (https://www.genomicsengland.co.uk/bioinformatics-partnership-with-illumina/). Other “interpretation partners” include Congenica, ICON, Fabric Genomics and WuxiNextCode (https://www.genomicsengland.co.uk/the-100000-genomes-project/data/current-research/). Further to this, industrial companies have been invited to engage with the project and access data by joining the Genomics Expert Network for Enterprises (GENE) for a fee.

Currently, novel or rare variants identified in rare disease patients in the 100,000 Genomes Project are “tiered” according to predicted pathogenicity, following the Association for Clinical Genetic Science's Best Practice Guidelines for Variant Classification (https://www.acgs.uk.com/quality/best-practice-guidelines/) which builds upon Standards and Guidelines for the Interpretation and Reporting of Sequence Variants in Cancer published by the Association for Molecular Pathology (Li et al., 2017). This allows classification of variants into Tier 1, variants with strong clinical significance; Tier 2, variants with potential clinical significance; Tier 3, variants of unknown clinical significance; and Tier 4, variants deemed benign or likely benign. Tiering is achieved using information from PanelApp, an online resource in which clinicians, academic researchers and laboratory scientists pool information about known disease genes, and pathogenic variants within them (https://panelapp.genomicsengland.co.uk/). This crowdsourcing tool enables a “virtual gene panel” approach to the analysis of genomic data; focusing on known or predicted pathogenic genes and variants. Patients' genomes are first analyzed against a panel of genes most closely associated with their disease phenotype (i.e., ciliopathy gene panels), then against other suitable gene panels with features overlapping the phenotype e.g., retinal dystrophy gene panel, neurology panel. Tier 1 variants are protein truncating (frameshift, stop gain, stop loss, splice acceptor variant, or splice donor variant) or *de novo* (protein truncating, missense, or splice region) variants in at least one transcript of a gene on the diagnostic grade “green” gene list in the virtual gene panel for the disorder in question. Tier 2 variants are protein altering variants, such as missense and splice region variants, in at least one transcript of a gene on the diagnostic grade “green” gene list in the virtual gene panel for the disorder in question. Tier 1 and 2 variants are not commonly found in the general healthy population, the allelic state matches the known mode of inheritance for the gene and disorder, and segregates with disease (where applicable). Protein truncating, *de novo* or protein altering variants affecting genes not in the virtual gene panel are Tier 3. If a variant does not meet any of these criteria it is untiered^1^.

To provide some sense of scale of the challenge faced, at the time of going to press, there were 2,605 Tier 3 variants in probands with the phenotype “primary ciliary disorders;” primary ciliary dyskinesia' and “primary ciliary dyskinesia::primary ciliary disorders.” Of these, 174 are frameshift/stop gain/stop loss/splice donor variant/splice acceptor variants i.e., presumed loss-of function, and 1,829 are missense variants, in around 115 genes. Similarly, at the time of going to press, there were 4,518 Tier 3 variants in probands with phenotype “Bardet-Biedl syndrome;” “Joubert syndrome” or “rare multisystem ciliopathy disorders.” Of these, 506 are frameshift/stop gain/stop loss/splice donor variant/splice acceptor variant i.e., presumed loss-of function, and 3,028 are missense variants. Characterizing the effect of these mutations poses a significant challenge in terms of computing power, manpower and bioinformatics expertise.

## Molecular MODELING of Ciliopathies

Traditional approaches to modeling non-motile ciliopathies involve 2D ciliated cell line cultures (Table 2) or whole animal studies, typically zebrafish (Marshall and Osborn, 2016; Song et al., 2016), mice (Norris and Grimes, 2012), *Xenopus* (Walentek and Quigley, 2017; Blum and Ott, 2018), chick (Schock et al., 2016) and *C.elegans* (Mok and Héon, 2012). Study of motile ciliopathies usually involves studying cells *in vivo*, or on *ex vivo* cultures such as mouse tracheal explants or nasal brush samples from patients with PCD, grown at the air-liquid interface (ALI) (Hirst et al., 2010). There are no adherent immortalized cell lines which can grow motile cilia, although a recent paper reported the immortalization of a multiciliated cell line with dyskinetic cilia (Kuek et al., 2018) which is unlikely to be useful as a control for studying motile ciliopathies. Much understanding of motile cilia has been achieved using single celled flagellated organisms such as *Chlamydomonas reinhardtii* (Harris et al., 2009) and *Trypanosoma brucei* (Langousis and Hill, 2014) which possess one and two flagella, respectively. More recent models include multiciliates planaria and paramecia (King and Patel-King, 2016; Fassad et al., 2018). These model systems offer numerous advantages, including ease of culture and biochemical purification of motile cilia and ease of genetic manipulation. For a useful review of cilia model organisms, see (Vincensini et al., 2011).

**Table 2:** Immortalized cell lines used for modeling ciliopathy mutations.

**Cell line name**	**Species origin**	**Tissue origin**	**Ciliation *in vitro***	**Transfection**	**ATCC ref**	**References**	**Notes**
A6	*Xenopus laevis*, frog, South African clawed	Kidney	^**^	?	CCL-102	Rafferty and Sherwin, 1969	Requires growth on porous collagen-coated filters to allow underside of cells to contact growth media. Grow very long cilia (up to 50 microns long). Can grow motile cilia.
ARPE-19	*Homo sapiens*	Retina—pigmented epithelium	^**^	^*^	CRL-2302	Dunn et al., 1998	
HEK293(T)	*Homo sapiens*	Embryonic kidney	^*^?	^****^	CRL-1573; CRL-6216	Graham et al., 1977	Often used as an exemplary transfection host cell line. Not well-characterized as being ciliated in culture, but cilia have been described on these cells. Requires growth on porous filters to allow underside of cells to contact growth media
HeLa	*Homo sapiens*	Cervix - adenocarcinoma	^*^	^***^	CCL-2	Jones et al., 1971	These are not well-characterized as being ciliated in culture, but cilia have been described on these cells.
hTERT-RPE1	*Homo sapiens*	Retina—pigmented epithelium	^**^	^**^	CRL-4000	Rambhatla et al., 2002	
LLC-PK1	*Sus scrofa* (pig)	Kidney—proximal tubule	^**^	?	CL-101	Perantoni and Berman, 1979	
mIMCD-3	*Mus musculus*	Kidney—inner medullary collecting duct	^***^	^***^	CRL-2123	Rauchman et al., 1993	
MDCK	*Canis familiaris*	Kidney—distal tubule/collecting duct	^**^	^**^	CCL-34	Gaush et al., 1966	Requires growth on porous filters to allow underside of cells to contact growth media.
NIH/3T3	*Mus musculus*	Fibroblast	^***^	^***^	CRL-1658	Jainchill et al., 1969	

*ATCC, American Type Culture Collection; ?, no information available. Asterisks indicate degree of ciliation achieved and ease of transfection in different cell lines*.

3D organoids derived from human embryonic stem (ES) cells or induced pluripotent stem cell (iPSCs) are increasingly replacing animals in ciliopathy research. These can be derived from patient fibroblasts, or can be genetically engineered to harbor patient mutations, to study effect of mutation and efficacy of possible treatments. In the past decade, techniques have particularly advanced in culture methods for producing *in vitro* 3D cell culture models to study cilia and ciliopathies. These include urine-derived renal epithelial cells (URECs) (Ajzenberg et al., 2015) and models of mammalian retina for studying retinal ciliopathies. Robust protocols for culture of retinal organoids from human embryonic stem (ES) cells and induced pluripotent stem cells (iPSCs) have been published and refined (Meyer et al., 2011; Kuwahara et al., 2015). These organoids form laminated neural retina with mature photoreceptor cells, which can be selectively isolated using specific cell surface markers (Lakowski et al., 2018). This provides a highly relevant human-derived model for studying retinal development and retinal degeneration. This is particularly useful for studying human retinal dystrophies which are not recapitulated in genetic mouse models, such as knock-in and knock-out mice models of RP associated with pre-mRNA splicing factor 31 mutations, which do show photoreceptor degeneration (Bujakowska et al., 2009). Similarly, culture techniques are advancing toward the ability to grow motile ciliated cells from iPSCs and ES cells, including in 3D spheroids (Firth et al., 2014; Konishi et al., 2016).

Techniques for genetically editing these ciliated cell models are also advancing, where primary patient cells are not suitable or available for research. This field is moving toward a point where investigators can replicate a potentially pathogenic variant of unknown clinical significance in a human ES cell or iPSC, and differentiate these cells toward a cell type relevant to the primary disease tissue to study the effect of mutation and evaluate methods of genetic correction. Since the development of zinc finger nucleases (ZFNs) (Bibikova et al., 2003) and transcription activator-like effector nucleases (TALENs) (Wood et al., 2011) it has been possible to edit the genome with a high degree of accuracy, to introduce DNA double strand breaks at specific genomic locations, which are generally repaired by error-prone non-homologous end joining (NHEJ) introducing small insertions or deletions, in order to create specific genetic knockouts. However, design and production of such zinc finger nucleases was slow and laborious. It is now possible to introduce such specific breaks with one common nuclease; Cas9 nuclease, significantly increasing throughput. Cas9 is targeted to the genome using a specific guide RNA, in a process termed Clustered Regularly Interspaced Short Palindromic Repeats (CRISPR)/Cas9 genome editing (Cong et al., 2013). The development of the “dual nickase” approach increases specificity and reduces off-target effects (Ran et al., 2013) but off-target effects remain one of the major challenges of scientists using CRISPR. Whilst knockouts can be generated at high efficiency, relying on NHEJ, the introduction of specific insertions, deletions, transitions or transversions using a specific repair template via homology directed repair (HDR), occurs at significantly lower frequency.

**Figure 3:**
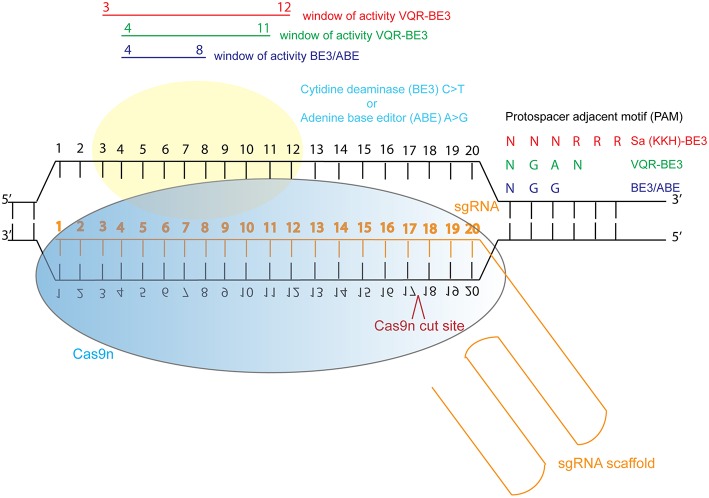
Schematic illustration of cytidine deaminase and adenine base editing of the genome. Abbreviations: sgRNA, single guide RNA; BE3, third generation base editor; ABE, adenine base editor.

In response to this, investigators have now developed base editing, a modified form of CRISPR/Cas9 genome editing, in which Cas9 nickase (Cas9n) is tethered to a cytidine deaminase enzyme which catalyzes the efficient conversion of C•G>T•A changes within a specific window of activity (Komor et al., 2016; Koblan et al., 2018). This refined genetic system can be used to study specific dominant, recessive and compound heterozygous mutations in relevant cells, or animal models. Recently, new adenine base editing tools have been developed, which enable engineering of specific A•T>G•C changes in cell lines (Gaudelli et al., 2017; Koblan et al., 2018). Both technologies have been optimized for mutation in mammalian systems (Koblan et al., 2018). C•G>T•A and A•T>G•C changes can now be efficiently engineered into cells and animals to model dominant disease. However, there is a restricted window of activity 4–8 nucleotides from the first nucleotide of the guide RNA, and guides can only be designed immediately upstream of a relevant protospacer adjacent motif (PAM). Engineering of Cas9 is enabling wider application of this base editing technology by adjusting the PAM sequence recognized by Cas9, so that with the newly engineered Cas9-cytidine deaminase fusions, approximately 2.5x more variants in ClinVar can be targeted using the technology (Kim et al., 2017) (Figure 3). Despite these current limitations, the technology is rapidly advancing and a “double hit” approach provides an efficient new method for modeling compound heterozygous mutations in cell systems. As most ciliopathies are recessive disorders, and compound heterozygosity is common in individuals from non-consanguineous unions, this provides a valuable tool for characterizing variants of unknown significance from the 100,000 Genomes Project.

Characterizing the genetics of ciliopathies may be a larger challenge than in other rare diseases, due to more complex genetics, for example the putative oligogenic inheritance in BBS (Katsanis et al., 2001) and modifier allele effects in multiple ciliopathies (Khanna et al., 2009; Davis et al., 2011; Cardenas-Rodriguez et al., 2013), but novel genome editing technologies provide solutions to these challenges. In order to process the large volume of variants, high-throughput approaches will need to be employed, for example high-content imaging screens such as previously published screens including a whole genome siRNA knockdown screen in ciliated mouse kidney cell line IMCD3 (Wheway et al., 2015) and a druggable genome siRNA knockdown screen in ciliated human retinal cell line hTERT-RPE1 (Kim et al., 2010).

## Health Potential and Future Opportunities of the 100,000 Genomes Project

Despite the challenges, there are undoubtedly enormous opportunities provided by this rich, varied and comprehensively phenotyped dataset. The project represents one of the greatest opportunities for novel disease gene discovery, especially in the case of very rare genes/genetic mutations. The aggregation of many families into the dataset allows multiple families with mutations in the same gene to be identified, leading to disease gene identification. One recent example is *PRPS1*, a gene in which heterozygous missense mutations were found to be carried by retinal dystrophy patients from 5 families in the 100,000 Genomes Project, providing robust genetic evidence for the cause of this condition. Mutations in *PRPS1* are normally found in the more severe Arts syndrome, Charcot-Marie Tooth, and nonsyndromic sensorineural deafness, so if mutations in this gene had only been identified in one family, this may have been disregarded as the pathogenic cause of disease (Fiorentino et al., 2018).

Furthermore, the aggregation of all rare disease patient data into one database may enable diagnosis of novel orphan ciliopathies in a way that was not previously possible. The extensive phenotypic and genotypic heterogeneity of ciliopathies raises the likelihood that there exist individuals or groups of individuals who have a disorder arising from a defect in cilia, but have not had their condition defined as a ciliopathy. The collection of all 60,000 rare disease individuals in the 100,000 Genomes Project may enable identification of novel, previously unrecognized ciliopathies.

**Figure 4:**
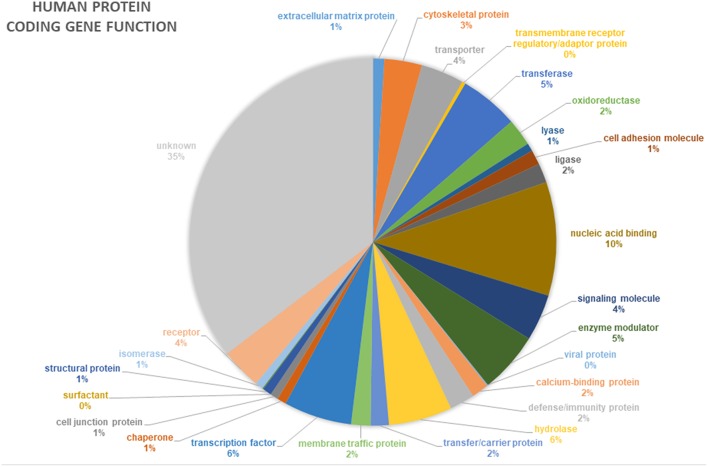
Proportion of annotated human protein coding genes with specific functions. Data from pantherdb.org. Thirty-five percentage of human genes have an unknown function.

In addition to novel disease gene discovery, the project offers many opportunities to gain novel insights into even the most basic gene functions. Around 35% of human genes still have no known function (pantherdb.org) (Figure 4). Functional genetics studies investigating genes and variants of interest from the 100,000 Genomes Project may uncover many novel developmental pathways and gene functionalities.

Such disease gene discoveries and new biological insights may provide opportunities for developing targeted therapies for ciliopathies, which currently have very few, if any, treatment options. A number of studies have investigated the efficacy and safety of gene therapy for treatment of ciliopathies, particularly in retinal ciliopathies. *RPGR*, mutations in which cause up to 60% of cases of X-linked RP (Vervoort et al., 2000) has been a particular focus for gene therapy development, including tested in large mammalian models, which have shown to be effective in preventing onset of degeneration (Beltran et al., 2012; Deng et al., 2015; Wu et al., 2015) and also successful in preventing progression of established disease (Beltran et al., 2015). More recently, *RPGR* gene editing approaches have been developed in addition to existing gene augmentation approaches (Deng et al., 2018). However, more than 20 years after the discovery of RPGR, there is still no gene therapy in clinic, although there are three clinical trials currently recruiting, at the time of writing (https://clinicaltrials.gov/ct2/results?cond=&term=rpgr&cntry=&state=&city=&dist=). There are now several more genes being targeted for gene therapy in retinal ciliopathies including *CEP290* (mutations in which cause LCA10) (Estrada-Cuzcano et al., 2012; Burnight et al., 2014; Zhang et al., 2018); and *LCA5* (Song et al., 2018). Gene therapy is also being investigated as a possible treatment for syndromic ciliopathies. Gene augmentation has been demonstrated to be effective and safe in *Bbs4* genetic mouse models of BBS; (McIntyre et al., 2012, 2013; Chamling et al., 2013), but the only clinical phenotype which was rescued was the olfactory sensory defect. It is clear that we need to look beyond gene therapy for treatment options for ciliopathies. The extensive genetic heterogeneity underlying these conditions is a hinderance to development of personalized medicines, but many recurrent and founder effect mutations are also found to underlie the ciliopathies. The future of effective drug development for ciliopathies requires an understanding of the molecular mechanism of disease, which requires genetic and cell biology studies that the 100,000 Genomes Project can accelerate. Much added value will arise from integration of genomics with multi-omics data (proteomics, transcriptomics, metabolomics, epigenomics) and deep phenotyping of ciliopathies, as recently discussed (Kenny et al., 2017).

Re-purposing and re-licensing of FDA-approved drugs could be the most rapid way to bring clinical therapeutics to patients. For example, it has been suggested that re-purposing of cyclin dependent kinase inhibitors could be an appropriate treatment option for a broad range of ciliopathies which have a common basis in replication stress (Slaats et al., 2015). Treatment with CDK inhibitors such as roscovitine has been shown to be effective in rescuing cilia defects in cells derived from patients with Joubert syndrome (Srivastava et al., 2017). Patients with mutations in *NEK1*, a DNA damage response gene mutated in patients with the ciliopathy short rib polydactyly syndrome (Thiel et al., 2011), could potentially be treated in this manner. NEK1 interacts with other ciliopathy proteins C21orf2 and SPATA7 (Wheway et al., 2015), suggesting that other ciliopathies could likewise be treated with CDK inhibitors. As a center for Hh signaling, cilia defects can also be potentially treated with Hh agonists (Hynes et al., 2014), for example purmorphamine, which has been shown to be effective in rescuing cilia defects in cells derived from patients with Joubert syndrome (Srivastava et al., 2017). Uncovering novel mechanisms of disease through functional characterization of variants in the 100,000 Genomes project ciliopathy patient dataset may well identify common pathways of disease which can be targeted to provide therapies for many individuals. Furthermore, if and when any potential therapies reach clinical trials, the national genomic registry provided by the 100,000 Genomes Project will allow rapid identification of individuals suitable for entering the trial.

Until treatments are developed, perhaps the main advantage of insights provided by the 100,000 Genomes Project is the ability to provide genetic diagnosis, for the purposes of better disease monitoring and management, carrier testing, and family planning. This is a particularly pressing need for rare, poorly recognized and difficult to diagnose conditions such as the ciliopathies. If a genetic diagnosis is made, family members can undergo carrier testing which can also inform marriage planning (Nouri et al., 2017; Komlosi et al., 2018). This is especially useful in communities practicing first cousin marriage (consanguinity) in which ciliopathies are at higher risk. One of the aims of the Human Genome Project in Saudi Arabia, where rates of first cousin marriage are around 40%, is to establish a service whereby every engaged couple can undergo whole genome sequencing to test for pathogenic alleles. Worldwide, couples carrying pathogenic variants can make informed decisions about planning pregnancies, including options for pre-implantation diagnosis (PGD). Genetic diseases require robust genetic information before they will be approved by the Human Fertilization and Embryology Authority (HFEA) for PGD. Many genetic subtypes of ciliopathies are now approved for PGD by HFEA, including BBS1, BBS10 and most genetic subtypes of Joubert syndrome (https://www.hfea.gov.uk/pgd-conditions/).

## Future Perspectives for UK Medical Genomics

On 31st September 2018, recruitment to the rare disease pathway of the 100,000 Genomes Project closed. DNA samples from 61,282 rare disease patients and family members have been deposited in the UK Biobank and 87,231 whole genome sequences have been produced, from rare disease and cancer patients.

Data from the project will continue to provide diagnoses and uncover novel biological insights for years to come. But what of the future of genomic testing in the NHS? In place of the project, from 1st October 2018 the new National Genomic Medicine Service was established, with an aim to provide consistent and equitable access to genomic testing services across the NHS. Genetic testing will now be conducted across a Genomic Laboratory Network of seven hubs, with choice of tests available dictated by the National Genomic Test Directory (https://www.england.nhs.uk/publication/national-genomic-test-directories/). Of the 190 rare diseases recruited into the project, neurological, neurodevelopmental conditions and complex congenital malformations, such as ciliopathies, are those most likely to continue to benefit from genome testing. There are now 22 indications for frontline WGS, 12 of which include ciliopathy phenotypes (Table 3). Bardet-Biedl syndrome and respiratory ciliopathies will be initially tested using WES or a large panel test, followed by WGS in year 3 if no results are found (Table 3).

**Table 3:** Conditions indicated for WGS diagnosis in the UK NHS.

**Clinical indication**	**Optimal family structure**	**Test method year 1**	**Test method year 2**	**Test method year 3**
Acutely unwell infants with a likely monogenic disorder	Trio	WGS	WGS	WGS
Ultra-rare and atypical monogenic disorders	Trio or singleton	WGS	WGS	WGS
Congenital malformation and dysmorphism syndromes—likely monogenic	Trio	WGS	WGS	WGS
Moderate, severe or profound intellectual disability	Trio	WGS	WGS	WGS
Floppy infant with a likely central cause	Trio	WGS	WGS	WGS
Skeletal dysplasia	Trio	WGS	WGS	WGS
Rare syndromic craniosynostosis or isolated multisuture synostosis	Trio	WGS	WGS	WGS
Neonatal diabetes	Trio	WGS	WGS	WGS
Likely inborn error of metabolism—targeted testing not possible	Trio	WGS	WGS	WGS
Hereditary ataxia with onset in adulthood	Singleton	WGS	WGS	WGS
Hereditary ataxia with onset in childhood	Trio or singleton	WGS	WGS	WGS
Early onset or syndromic epilepsy	Trio	WGS	WGS	WGS
Childhood onset hereditary spastic paraplegia	Trio or singleton	WGS	WGS	WGS
Arthrogryposis	Trio	WGS	WGS	WGS
Other rare neuromuscular disorders	Trio or singleton	WGS	WGS	WGS
Cerebellar anomalies	Trio	WGS	WGS	WGS
Holoprosencephaly—NOT chromosomal	Trio	WGS	WGS	WGS
Hydrocephalus	Trio	WGS	WGS	WGS
Cerebral malformation	Trio	WGS	WGS	WGS
Severe microcephaly	Trio	WGS	WGS	WGS
Childhood onset leukodystrophy	Trio	WGS	WGS	WGS
Cystic renal disease	Singleton	WGS	WGS	WGS
Parental sequencing for lethal autosomal recessive disorders	Parents only	WES	WES	WGS
Bardet-Biedl syndrome	Singleton	WES or large panel	WES or large panel	WGS
Fetal anomalies with a likely genetic cause	Singleton	WES or large panel	WES or large panel	WGS
Hypertrophic cardiomyopathy—teen and adult	Singleton	WES or large panel	WES or large panel	WES or large panel
Dilated cardiomyopathy—teen and adult	Singleton	WES or large panel	WES or large panel	WES or large panel
Molecular autopsy	Singleton	WES or large panel	WES or large panel	WES or large panel
Progressive cardiac conduction disease	Singleton	WES or large panel	WES or large panel	WES or large panel
Thoracic aortic aneurysm or dissection	Singleton	WES or large panel	WGS	WGS
Pediatric or syndromic cardiomyopathy	Singleton	WES or large panel	WES or large panel	WGS
Primary lymphoedema	Singleton	WES or large panel	WES or large panel	WGS
Non-syndromic hearing loss	Singleton	WES or large panel	WES or large panel	WGS
Monogenic diabetes	Singleton	WES or large panel	WES or large panel	WES or large panel
Growth failure in early childhood	Singleton	WES or large panel	WGS	WGS
Bilateral congenital or childhood onset cataracts	Singleton	WES or large panel	WGS	WGS
Retinal disorders	Singleton	WES or large panel	WES or large panel	WGS
Structural eye disease	Singleton	WES or large panel	WES or large panel	WGS
Cholestasis	Singleton	WES or large panel	WES or large panel	WES or large panel
Disorders of sex development	Singleton	WES or large panel	WGS	WGS
Possible X-linked retinitis pigmentosa	Singleton	WES or large panel	WES or large panel	WGS
Sorsby retinal dystrophy	Singleton	WES or large panel	WES or large panel	WGS
Doyne retinal dystrophy	Singleton	WES or large panel	WES or large panel	WGS
Polycystic liver disease	Singleton	WES or large panel	WES or large panel	WES or large panel
Infantile inflammatory bowel disease	Singleton	WES or large panel	WES or large panel	WES or large panel
Bleeding and platelet disorders	Singleton	WES or large panel	WES or large panel	WGS
Rare anemia	Singleton	WES or large panel	WES or large panel	WGS
Cytopenia—NOT Fanconi anemia	Singleton	WES or large panel	WES or large panel	WGS
Cytopenia—Fanconi breakage testing indicated	Singleton	WES or large panel	WES or large panel	WGS
Thrombophilia with a likely monogenic cause	Singleton	WES or large panel	WES or large panel	WES or large panel
Primary immunodeficiency	Singleton	WES or large panel	WGS	WGS
Glycogen storage disease	Singleton	WES or large panel	WES or large panel	WES or large panel
Lysosomal storage disorder	Singleton	WES or large panel	WES or large panel	WES or large panel
Mitochondrial DNA maintenance disorder	Singleton	WES or large panel	WGS	WGS
Mitochondrial disorder with complex I deficiency	Singleton	WES or large panel	WGS	WGS
Mitochondrial disorder with complex II deficiency	Singleton	WES or large panel	WGS	WGS
Mitochondrial disorder with complex III deficiency	Singleton	WES or large panel	WGS	WGS
Mitochondrial disorder with complex IV deficiency	Singleton	WES or large panel	WGS	WGS
Mitochondrial disorder with complex V deficiency	Singleton	WES or large panel	WGS	WGS
Possible mitochondrial disorder—nuclear genes	Singleton	WES or large panel	WGS	WGS
Ehlers Danlos syndrome with a likely monogenic cause	Singleton	WES or large panel	WES or large panel	WES or large panel
Osteogenesis imperfecta	Singleton	WES or large panel	WES or large panel	WES or large panel
Adult onset dystonia, chorea or related movement disorder	Singleton	WES or large panel	WES or large panel	WGS
Childhood onset dystonia, chorea or related movement disorder	Singleton	WES or large panel	WES or large panel	WGS
Adult onset neurodegenerative disorder	Singleton	WES or large panel	WGS	WGS
Adult onset hereditary spastic paraplegia	Singleton	WES or large panel	WGS	WGS
Adult onset leukodystrophy	Singleton	WES or large panel	WGS	WGS
Paroxysmal neurological disorders, pain disorders and sleep disorders	Singleton	WES or large panel	WES or large panel	WGS
Hereditary neuropathy—NOT PMP22 copy number	Singleton	WES or large panel	WGS	WGS
Congenital muscular dystrophy	Singleton	WES or large panel	WGS	WGS
Congenital myaesthenic syndrome	Singleton	WES or large panel	WGS	WGS
Congenital myopathy	Singleton	WES or large panel	WGS	WGS
Limb girdle muscular dystrophy	Singleton	WES or large panel	WGS	WGS
Neuromuscular arthrogryposis	Singleton	WES or large panel	WGS	WGS
Cerebral vascular malformations	Singleton	WES or large panel	WES or large panel	WES or large panel
Renal tubulopathies	Singleton	WES or large panel	WES or large panel	WES or large panel
Nephrocalcinosis or nephrolithiasis	Singleton	WES or large panel	WES or large panel	WES or large panel
Unexplained pediatric onset end-stage renal disease	Singleton	WES or large panel	WES or large panel	WES or large panel
Proteinuric renal disease	Singleton	WES or large panel	WGS	WGS
Laterality disorders and isomerism	Singleton	WES or large panel	WES or large panel	WES or large panel
Respiratory ciliopathies including non-CF bronchiectasis	Singleton	WES or large panel	WES or large panel	WES or large panel
Epidermolysis bullosa and congenital skin fragility	Singleton	WES or large panel	WES or large panel	WES or large panel
Neonatal erythroderma	Singleton	WES or large panel	WES or large panel	WES or large panel

This represents the largest restructuring of genetic testing in the history of the NHS and serves as a model for other countries embarking on similar initiatives. These clinical advances will be of enormous benefit to patients with complex syndromic conditions such as ciliopathies. The aim is that WGS will now be the standard, frontline diagnostic test for many of these families, and hopefully increases in diagnostic rates beyond 60% will be start to be achieved. The medical benefits to families of achieving a rapid diagnosis through WGS for rare diseases like ciliopathies and the costs saved to the NHS can be calculated (Mestek-Boukhibar et al., 2018). Studies in the USA have shown that WGS is by far the most time- and cost-effective way to achieve a diagnosis for complex, heterogeneous disorders such as ciliopathies. In the case of general neurodevelopmental disorders, which are also extensively heterogeneous, WGS can reduce time to diagnosis by 77 months, reduce cost of diagnosis by $11,460, and improve clinical care (Soden et al., 2014). For extremely genetically heterogeneous conditions such as neurodevelopmental disorders and ciliopathies, WGS provides an opportunity to allow families to circumvent the traditional “diagnostic odyssey” (Sawyer et al., 2016). Genomics England now plan to sequence 5 million genomes over next 5 years.

## Global Perspective and Future Challenges

There are many other national genome projects around the world, including many with a focus on understanding rare disease, cancer, and genomics for precision medicine. Launched just after the UK's 100,000 Genomes Project, in December 2013, the Saudi Human Genome Program aims to sequence 100,000 individuals, with a specific focus on understanding rare inherited disease, common in Saudi Arabia where first-cousin marriage is common. Two of the largest national genome projects at the time of writing are the USA's $215 million Precision Medicine Initiative announced in January 2015, which aims to sequence 1 million genomes (https://www.nature.com/news/us-precision-medicine-proposal-sparks-questions-1.16774) and China's genomic medicine initiative, launched in 2016, which also aims to sequence 1 million genomes, as part of its 13^th^ five-year plan. Two of the longest running are the Estonia Genome Project (since 2001) and DeCODE project in Iceland, which has been running since 1996 and has sequenced 160,000 individuals, a large proportion of the entire Icelandic population. Others include Genome Russia Project, Proyecto Genoma Navarra (NAGEN) (Spain), Qatar Genome Programme, Belgian Medical Genomics Inititative. More recently, in 2016, France announced its Genomic Medicine Plan 2025 “Médecine Génomique 2025' which involves cooperation with Genomics England, to co-fund analysis of genomes. More local efforts which are large-scale in terms of numbers, but are not nationwide include Geisinger MyCode (Pennsylvania and New Jersey) which has recruited 190,000 patients, and the Utah Genome Project. Transnational efforts include GenomeAsia100K and international efforts include the Personal Genome Project. Transnational and international collaborative efforts may become more commonplace in the future, to increase the power of shared resources.

As well as state-funded clinical and research genomics projects, there also exist privately funded genomics projects, including Craig Venter's “Human Longevity,” which aims to sequence 1 million genomes by 2020. AstraZeneca's genomics initiative (in partnership with Human Longevity, Genomics England, Montreal Heart Institute, Sanger Institute and University of Helsinki) uses retrospective patient data from clinical trials over the past 10 years, and will continue to collect data until 2026. In total, up to 2 million genomes will be sequenced, including 500,000 from patients who have taken part in AstraZeneca clinical trials, to significantly enhance drug development for precision medicine approaches.

Each project has different specific aims but they each share many similarities. They all use Illumina short-read sequencing technologies, although it seems likely that in the future at least some will couple these with longer-read sequencing using platforms such as Pacific Biosciences (PacBio) and Oxford Nanopore. Most projects are driven by the same financial initiatives. Since 2008, there has been a global concerted effort to reduce national spending on healthcare, which in developed countries typically accounts for spending equivalent to 10–15% of national GDP (http://www.abpi.org.uk/facts-and-figures/global-pharmaceutical-market/global-health-expenditure-as-a-share-of-gdp/). The Saudi Human Genome Program estimates the total global annual cost of treating rare inherited disease at around US$27 billion (Project Team, 2015). A substantial reduction in children born with genetic disabilities, as a result of improved genetic diagnostics through understanding gained from genome projects, could immediately save over US$270 million. Furthermore, most governments cite the importance of remaining, or becoming, internationally competitive in the nascent field of genome-guided drug development as reasons behind their investment in large-scale genomics projects. The pharmaceutical industry is one of the world's largest industries, worth in excess of $800,000 million annually (http://www.abpi.org.uk/facts-and-figures/global-pharmaceutical-market/top-10-pharmaceutical-markets-by-value-usd/).

In order to achieve the greatest global health benefits from the information in these multiple genomics projects, responsible and secure data sharing is essential. Achieving this whilst maintaining patient confidentiality and data security is a global challenge. The Global Alliance for Genomics and Health (GA4GH) was established in 2013 to develop best-practice guidelines, and develop tools for genome data sharing. Most recently, GA4GH have developed the application programming interface (API) Beacon, which allows organizations to share genomic and health data in a way which preserves patient confidentiality and data anonymity. Through Beacon, individuals who are not registered and approved users of individual genomics datasets, such as a GeCIP member, can still query these datasets to retrieve information about specific alleles. This saves time; there is no need to go through an often lengthy database access protocol and it actually preserves patient confidentiality since no raw genome data is accessed. Beacon is part of the new interoperability standards, contributing to the Framework for Responsible Sharing of Genomic and Health-Related Data developed by GA4GH (https://www.ga4gh.org/genomic-data-toolkit/regulatory-ethics-toolkit/framework-for-responsible-sharing-of-genomic-and-health-related-data/). Congenica and Genomics England are members of the GA4GH and contribute to the Steering Committee of GA4GH, so it seems likely that at some point in the future data will become more widely available for exploitation for the benefit of human health.

Meaningful and effective sharing of data from many genome projects also poses a practical challenge in terms of interoperability of analytical platforms. Whilst all large scale genome projects predominantly use Illumina sequence technologies, each program has bespoke data standards, analytical pipelines, storage and access protocols which are potentially incompatible with each other. There may also be variations in the standards applied to use of phenotypic terms, variant calling and descriptions of pathogenicity of alleles. GA4GH develops tools and defines standards to harmonize data generation, analysis, storage and access. Recently, GA4GH developed a set of standards for variant calling, which allows stratification of performance by variant type and genome context (Krusche et al., 2018). Furthermore, GA4GH develops standards for workflow development, and APIs for packaging of workflows to support their application within multiple different environments. This will allow researchers to run the same analysis workflow on data in multiple clouds and environments in order to standardize data analysis across projects.

Ensuring interoperability of platforms will becoming increasingly important as more genome projects incorporate long-read sequence technologies to complement short-read sequence technologies. This approach allows resolution and alignment of repetitive regions, including regions with many short tandem repeats, or genes with many pseudogenes, phasing of compound heterozygous mutations and provides information about epigenetic marks (Simpson et al., 2017; Pollard et al., 2018). This has clinical relevance in ciliopathies; ADPKD associated with *PKD1* mutations is difficult to diagnose genetically due to 6 pseudogenes of *PKD1* on chromosome 16 with 97.7% sequence identity (Eisenberger et al., 2015). Similarly, in the X-linked retinal ciliopathy retinitis pigmentosa, diagnostic yields are low due to the fact that most patients have a mutation in the highly repetitive region of *RPGR* ORF15, which is difficult to amplify by PCR and Sanger sequencing, and difficult to align with Illumina short read sequencing (Vervoort et al., 2000; Branham et al., 2012).

As well as ensuring integration of different sequencing analysis platforms, it is essential to ensure that platforms for sequence storage and analysis integrate properly with electronic clinical data management systems. This is particularly important for reporting and genetic counseling, and clinical management of patients in receipt of a genetic diagnosis (Welch et al., 2018). It is also crucial to the meaningful analysis of genome data, in the context of detailed medical records including family history, phenotype (using standard HPO terms) and treatment history. GA4GH have developed best practice guidelines for using Family Health History patient record systems with genomics data.

Complex challenges remain, including standardizing a definition of what constitutes a pathogenic allele across all genomics projects. The Association for Clinical Genetic Science's Best Practice Guidelines for Variant Classification 2017 (Ellard et al., 2017) forms the basis of the standard for the 100,000 Genomes Project, based upon Standards and Guidelines for the Interpretation and Reporting of Sequence Variants in Cancer published by the Association for Molecular Pathology (Li et al., 2017). However, it must be acknowledged that we remain relatively naïve in our understanding of pathogenicity, particularly with regard to variants affecting splicing. It has been calculated that 35-40% of pathogenic variants in non-canonical splice site positions are missing from public databases, suggesting that we currently under-diagnose pathogenic causes of disease associated with splicing (Lord et al., 2018). We do not yet know enough about the functional effect of non-coding variants, subtle missense variants and variants in exonic splice enhancers to be able to report these back to patients without functional validation. This is likely to be the significant bottleneck in exploitation of the expanding wealth of genome and population-genetics data. Most *in silico* predictors of pathogenicity are notoriously unreliable, and will only improve with machine learning aided by robust *in vitro* data from functional experiments. This is time-consuming and expensive work. Beyond simple monogenic disorders, we also need frameworks for consistently assigning pathogenicity based on polygenic risk scores, and combined total gene variation scores (Khera et al., 2018; Mossotto et al., 2018). For these reasons, it seems appropriate that this remains an ongoing area of development by GA4GH's Variation Annotation Task Team.

## Global Social Challenges

The UK, USA and China all strive to be global leaders in this area, and as a result, have each launched ambitious genome sequencing projects such as the 100,000 Genomes Project, arguably before the necessary skills, understanding or, importantly, public appetite, to manage these projects effectively is in place. Public understanding and trust of genomics remains a global challenge to the success of all genomics projects. Lack of understanding or confidence in genomics in the workforce and the patient population poses a barrier to effective implementation and recruitment to genomics projects. This requires an entire “Workforce transformation” (https://www.genomicseducation.hee.nhs.uk/news/item/357-transforming-healthcare-the-impact-of-genomics-on-the-nhs/). Health Education England, (HEE) is a key delivery partner in the 100,000 Genomes Project and has developed a Genomics Education Programme delivered by higher education providers around the UK as a postgraduate masters (MSc) in Genomic Medicine. Parts of this programme are also possible to study, with a shorter training period, awarded as a PGCert and PGDip, or as standalone modules available as part of continuing professional development (CPD). Courses are designed to fit around work, or to be attended part-time, by staff within the NHS and a One-day Primer In Genomic Medicine is currently offered by University of Southampton (https://www.southampton.ac.uk/medicine/primer-in-genomic-medicine-web-form.page; https://www.genomicseducation.hee.nhs.uk/taught-courses/courses/primer-genomic-medicine/.

Patient education and education of the general public in genomics remains a barrier to greater success and higher recruitment, and impacts on truly “*informed* consent.” Furthermore, the nature of a consent process in which all individuals must consent to the research element of the project, which includes sharing of information with commercial companies, raises issues around autonomy and consent (Dheensa et al., 2018). The public outreach initiative “Socializing the Genome,” developed by West of England Genomic Medicine Center, reached 19,000 people across the south-west, now launched across Manchester region (https://www.genomicseducation.hee.nhs.uk/news/item/347-how-lego-pro-bots-are-bringing-genomics-to-life-in-the-classroom/). At a time when public understanding, including in the workforce, remains low, there is concern that mainstreaming of genomics services at this point in time may be premature (Ormondroyd et al., 2018). Ethical implications of incidental findings, data security and anonymity, and intellectual property of findings resulting from genome studies also remain unresolved issues across all projects. Many professionals currently believe incidental findings should be reported with caution, with an “approach that is responsive to accumulating evidence.” These issues present a significant challenge (Ormondroyd et al., 2018). The general consensus is that there is not enough evidence to form a robust policy regarding secondary findings yet, so data should be interpreted with caution (Ormondroyd et al., 2018). Similarly, decisions about how often to reanalyze data and the mechanism for reporting data back to patients still requires resolution.

It is of utmost importance for healthcare professionals, researchers, and policymakers to be open and honest about the challenges, in order to avoid “genohype” and setting unrealistic expectations. In the past there has been a tendency to overstate the opportunities and understate the risks associated with genetic testing (Samuel and Farsides, 2017) and this has led to some loss of public confidence in genetics. Time taken to return findings remains a significant issue in genomic testing (Moss and Wernham, 2018). Transparency, clear and ongoing communication between healthcare professionals and patients is essential (Dheensa et al., 2018), communicating the possibility of no certain findings either now, or in the near future. We also need to reconsider our concept of the need for fully informed consent, which may not be possible in genomics. Rather, we must consider the general social contract around healthcare provision in the UK, and commitments to public education in the area of genomics.

## Concluding Remarks

The 100,000 Genomes Project provides many opportunities and challenges to improve our understanding of ciliopathies. Perhaps the real power in this dataset will be realized through aggregation of this information with other genome projects worldwide. For this to be a success, data security, regulation and ethics must be at the center of such efforts, to establish and maintain public trust in genome testing. There must be a truly global effort to standardize phenotypic descriptions, data standards, analysis pipelines and mechanisms for data sharing and discovery. Perhaps then, with global cooperation, we can finally increase diagnostic yields, inform better clinical management and translate new understanding into targeted therapies for ciliopathy patients.

## Data Availability

All datasets generated for this study are included in the manuscript and/or the supplementary files.

## Author Contributions

GERC provided essential data for this publication. All authors listed have made a substantial, direct and intellectual contribution to the work, and approved it for publication.

### Conflict of Interest Statement

The authors declare that the research was conducted in the absence of any commercial or financial relationships that could be construed as a potential conflict of interest.

## Footnotes

^1^For further information we direct readers to https://panelapp.genomicsengland.co.uk/
