# Corrigendum: Opportunities and Challenges for Molecular Understanding of Ciliopathies–The 100,000 Genomes Project

**DOI:** 10.3389/fgene.2019.00569

**Published:** 2019-08-13

**Authors:** Gabrielle Wheway, Genomics England Research Consortium, J. C. Ambrose, Hannah M. Mitchison

**Affiliations:** (1) Genomics England, London, UK; (2) William Harvey Research Institute, Queen Mary University of London, London, EC1M 6BQ, UK; ^1^Human Development and Health, Faculty of Medicine, University of Southampton, Southampton General Hospital, Southampton, United Kingdom; ^2^Genetics and Genomic Medicine, University College London, UCL Great Ormond Street Institute of Child Health, London, United Kingdom

**Keywords:** 100,000 Genome Project, ciliopathies, cilia, genomics, genetics

In the original article, there was an error. We incorrectly stated that ‘If a predicted pathogenic variant is found in the primary [gene] panel [on PanelApp], this will be classified as Tier 1. If a predicted pathogenic variant is found in another associated panel, this will be classified as Tier 2. If a predicted pathogenic variant in any other gene is found, this will be classified as Tier 3'.

A correction has been made to the article section **Ciliopathy Genomics Data Analysis in the 100,000 Genomes Project**, Paragraph 2.

“Currently, novel or rare variants identified in rare disease patients in the 100,000 Genomes Project are “tiered” according to predicted pathogenicity, following the Association for Clinical Genetic Science's Best Practice Guidelines for Variant Classification (https://www.acgs.uk.com/quality/best-practice-guidelines/) which builds upon Standards and Guidelines for the Interpretation and Reporting of Sequence Variants in Cancer published by the Association for Molecular Pathology (Li et al., 2017). This allows classification of variants into Tier 1, variants with strong clinical significance; Tier 2, variants with potential clinical significance; Tier 3, variants of unknown clinical significance; and Tier 4, variants deemed benign or likely benign. Tiering is achieved using information from PanelApp, an online resource in which clinicians, academic researchers and laboratory scientists pool information about known disease genes, and pathogenic variants within them (https://panelapp.genomicsengland.co.uk/). This crowdsourcing tool enables a “virtual gene panel” approach to the analysis of genomic data; focusing on known or predicted pathogenic genes and variants. Patients' genomes are first analyzed against a panel of genes most closely associated with their disease phenotype (i.e., ciliopathy gene panels), then against other suitable gene panels with features overlapping the phenotype e.g., retinal dystrophy gene panel, neurology panel. Tier 1 variants are protein truncating (frameshift, stop gain, stop loss, splice acceptor variant, or splice donor variant) or *de novo* (protein truncating, missense, or splice region) variants in at least one transcript of a gene on the diagnostic grade “green” gene list in the virtual gene panel for the disorder in question. Tier 2 variants are protein altering variants, such as missense and splice region variants, in at least one transcript of a gene on the diagnostic grade “green” gene list in the virtual gene panel for the disorder in question. Tier 1 and 2 variants are not commonly found in the general healthy population, the allelic state matches the known mode of inheritance for the gene and disorder, and segregates with disease (where applicable). Protein truncating, *de novo* or protein altering variants affecting genes not in the virtual gene panel are Tier 3. If a variant does not meet any of these criteria it is untiered.”

For further information we direct readers to https://panelapp.genomicsengland.co.uk/.

We wish to extend our thanks to Prof Sian Ellard, South West NHS Genomic Medicine Centre, for bringing this to our attention.

**Additionally, in the original article there was another error. Genomics England** was not included as an author in the published article. The corrected Author Contributions Statement appears below.

“GERC provided essential data for this publication. All authors listed have made a substantial, direct and intellectual contribution to the work, and approved it for publication.”

Additionally, the consortium members have now been added to the Acknowledgments. The corrected Acknowledgment section appear below:

“GW was supported by a Wellcome Trust Seed Award in Science (204378/Z/16/Z) and a University of Southampton Faculty of Medicine Research Management Committee Research Project Award. HM was supported by Great Ormond Street Children's Charity grant Leadership awards (V1299, V2217), the NIHR Biomedical Research Center at Great Ormond Street Hospital for Children NHS Foundation Trust and University College London and the COST Action BEAT-PCD: Better Evidence to Advance Therapeutic options for PCD network (BM1407). This research was made possible through access to the data and findings generated by the 100,000 Genomes Project. The 100,000 Genomes Project is managed by Genomics England Limited (a wholly owned company of the Department of Health). The 100,000 Genomes Project is funded by the National Institute for Health Research and NHS England. The Wellcome Trust, Cancer Research UK and the Medical Research Council have also funded research infrastructure. The 100,000 Genomes Project uses data provided by patients and collected by the National Health Service as part of their care and support.”

The authors apologize for this error and state that this does not change the scientific conclusions of the article in any way. The original article has been updated.
